# Impact of day hospital care on adherence to psychiatric follow-up appointments and medications in patients with delusional disorder

**DOI:** 10.1192/j.eurpsy.2021.1269

**Published:** 2021-08-13

**Authors:** A. González-Rodríguez, N. Sanz, A. Guàrdia, A. Alvarez Pedrero, D. Garcia Pérez, G.F. Fucho, L. Delgado, I. Parra Uribe, J.A. Monreal, D. Palao Vidal, J. Labad

**Affiliations:** 1 Mental Health, Parc Taulí University Hospital. Autonomous University of Barcelona (UAB). I3PT, Sabadell, Spain; 2 Department Of Mental Health, Parc Taulí University Hospital. Autonomous University of Barcelona (UAB). I3PT. CIBERSAM, Sabadell, Spain; 3 Mental Health, Hospital of Mataró. Consorci Sanitari del Maresme. CIBERSAM., Mataró, Spain

**Keywords:** Day hospital, Delusional disorder, adherence, psychosis

## Abstract

**Introduction:**

Day care programs have been extensively used to treat people with acute psychiatric disorders. Day hospitals (DH) can act as an alternative to admission in patients with acute symptoms, shorten the duration of admission, be useful for rehabilitation and maintenance care or enhance treatment in patients with poor adherence to outpatient care. Few research has been conducted in delusional disorder (DD).

**Objectives:**

To investigate whether DH care increases adherence with psychiatric appointments in patients with DD. To describe functions of partial hospitalization in DD.

**Methods:**

Comparative study including DD patients who attended a DH (Group 1:n=12) versus patients who did not receive DH care (Group 2;n=7). Patients attending DH were classified into 3 groups according to the program function at referral. Adherence with outpatient follow-up appointments (primary outcome) and pharmacy refill data (secondary outcome) were assessed after discharge over a 6-month period (DH) and compared with group 2. For statistical analyses, non-parametric tests were performed.

**Results:**

Program function (DH): alternative to admission (n=4); shortening of admission (n=5) and enhancing outpatient treatment (n=3). Patients receiving DH care were more frequently referred from the inpatient unit or emergency department compared to those who did not attend DH (commonly referred from primary care services). No statistically significant differences were found between both groups in adherence to psychiatric appointments. Patients who attended DH showed higher compliance with antipsychotics (89.29% vs.72.62, p<0.05).

**Conclusions:**

DH care may be a useful alternative to increase adherence with antipsychotics in DD patients with poor awareness of illness.

**Conflict of interest:**

AGR has received honoraria, registration for congresses and/or travel costs from Janssen, Lundbeck-Otsuka and Angelini.

